# Clinical Presentation and Outcomes of Antineutrophil Cytoplasmic Autoantibody–Negative Pauci-Immune Glomerulonephritis

**DOI:** 10.1016/j.ekir.2025.02.032

**Published:** 2025-03-03

**Authors:** Lauren Floyd, Anamay Shetty, Adam D. Morris, Krešimir Galešić, Mohamed Elsayed, Grace Lavery, Amrita Dhutia, Sorcha O’Brien, Sinead Stoneman, Allyson Egan, Mark A. Little, Vojtech Kratky, Zdenka Hruskova, Vladimir Tesar, Marek Kollar, Anke von Bergwelt-Baildon, Ulf Schönermarck, Eveline Y. Wu, Lauren Blazek, Vimal K. Derebail, Mariam Al-Attar, Nina Brown, Beatriz Sánchez Álamo, Wing-Yin Leung, Bryan Chang, Maria Letizia Urban, Federico Alberici, Luis F. Quintana, Oliver Flossmann, Silke R. Brix, Duvuru Geetha, Stephen McAdoo, Ajay P. Dhaygude, Andreas Kronbichler, Matija Crnogorac

**Affiliations:** 1 Division of Cardiovascular Sciences, The University of Manchester, Manchester, UK; 2 Renal Department, Royal Preston Hospital, Lancashire Teaching Hospitals, UK; 3 Oxford University Hospitals NHS Foundation Trust, UK; 4 Department of Nephrology and Dialysis, Dubrava Clinical Hospital, Zagreb, Croatia; 5 Imperial College Renal and Transplant Centre, Hammersmith Hospital, London, UK; 6 Trinity Kidney Centre, Trinity Translational Medicine Institute, Trinity College, Dublin, Ireland; 7 Department of Renal Medicine, Cork University Hospital, Cork, Ireland; 8 Trinity Health Kidney Centre, Tallaght University Hospital, Dublin, Ireland; 9 Department of Nephrology, General University Hospital in Prague and First Faculty of Medicine, Charles University, Prague, Czech Republic; 10 Department of Pathology, Institute of Clinical and Experimental Medicine, Prague, Czech Republic; 11 Nephrology Division, Department of Medicine IV, LMU University Hospital, LMU Munich, Germany; 12 Department of Pediatrics, Division of Allergy, Immunology, and Rheumatology, University of North Carolina at Chapel Hill, Chapel Hill, North Carolina, USA; 13 UNC Kidney Center, Division of Nephrology and Hypertension, Department of Medicine, University of North Carolina at Chapel Hill, Chapel Hill, North Carolina, USA; 14 Renal Department, Salford Royal Hospital, Northern Care Alliance NHS Foundation Trust, UK; 15 Department of Nephrology, Hospital Universitario Ramón y Cajal, Madrid, Spain; 16 Renal, Transplantation and Urology Unit, Manchester University NHS Foundation Trust, UK; 17 Department of Medicine, University of Cambridge, Cambridge, UK; 18 Department of Experimental and Clinical Medicine, University of Florence, Firenze, Italy; 19 Department of Medical and Surgical Specialties, University of Brescia, Brescia, Italy; 20 Department of Nephrology and Renal Transplantation, Hospital Clínic de Barcelona, University of Barcelona, IDIBAPS, Spain; 21 Department of Nephrology, Royal Berkshire Hospital, Reading, Berkshire, UK; 22 Division of Cell Matrix Biology and Regenerative Medicine, The University of Manchester, Manchester, UK; 23 Division of Nephrology, Johns Hopkins University School of Medicine, Baltimore, USA; 24 Department of Internal Medicine, Nephrology and Hypertension, Medical University Innsbruck, Austria

**Keywords:** ANCA-associated vasculitis, ANCA-negative, end-stage kidney disease, kidney biopsy, pauci-immune glomerulonephritis, vasculitis

## Abstract

**Introduction:**

Antineutrophil cytoplasmic autoantibody (ANCA)-associated vasculitis is a rare, complex autoimmune condition. Although ANCAs have a pathogenic role, they are considered a suboptimal biomarker of disease activity. Previous studies suggest differences in clinical phenotypes and outcomes in those without detectable circulating autoantibody. This study aimed to investigate the clinical presentation, histopathological findings, treatment practices, and outcomes of patients with ANCA-negative pauci-immune glomerulonephritis (PIGN).

**Methods:**

A retrospective, multicenter cohort study was conducted from 2002 to 2022 and included those with biopsy-proven PIGN. We aimed to investigate differences in presentation, clinical outcomes, and treatment practices of patients with ANCA-negative PIGN when compared with ANCA-positive controls.

**Results:**

In total, 132 ANCA-negative and 127 ANCA-positive patients were included. ANCA-negative patients were younger (*P* < 0.001), more commonly presented with renal-limited disease (*P* < 0.001), had worse estimated glomerular filtration rate at diagnosis (*P* < 0.02) and higher rates of proteinuria (*P* < 0.01). Controlling for age, sex, ethnicity, and recruiting center, ANCA-negative patients had lower rates of relapse (*P* < 0.001) and higher rates of end-stage kidney disease (ESKD) at 1 and 3 years (*P* < 0.001). Standard remission induction and maintenance therapies were used less often in ANCA-negative patients.

**Conclusion:**

The precise pathophysiology and factors contributing to the clinical phenotype of ANCA-negative PIGN remain unclear and potentially represent a distinct disease entity. Adverse outcomes may result from delays in diagnosis, advanced disease at presentation, and less intense immunosuppressive treatment. Current classification criteria inadequately address ANCA-negative disease and collaborative research, which includes ANCA-negative patients in trials is needed.

## Graphical abstract

**Figure:**
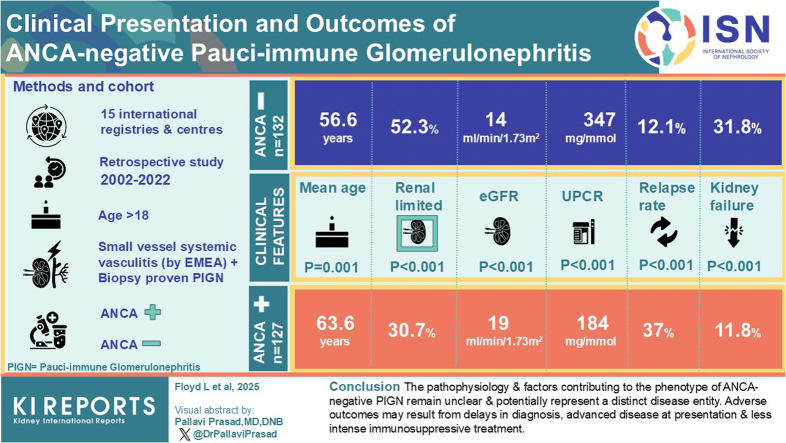

ANCA-associated vasculitis is a rare and complex autoimmune condition. It is characterized by inflammation and necrosis of small vessels, which can lead to multiorgan failure. Kidney involvement, often referred to as PIGN, is highly prevalent and histologically characterized by focal necrotizing and crescentic glomerular lesions in the absence of significant immune deposits.^1^^,^^2^

The current known pathogenesis of ANCA-associated vasculitis is intricately linked to circulating ANCAs categorized into perinuclear and cytoplasmic staining patterns.^3^ These ANCAs target 2 key antigens; myeloperoxidase (MPO) and proteinase 3 (PR3). However, a significant proportion of patients develop PIGN in the absence of detectable circulating ANCAs. Additional research has shown other immunological factors such as neutrophil extracellular trap generation, complement activation, and endothelial cell damage play a role in the pathogenesis of PIGN.^4^ Although ANCAs have a pathogenic role in disease, they are considered a suboptimal biomarker of disease activity and the absence or disappearance is not a prerequisite for disease activity or remission.

Seronegative ANCA-associated vasculitis has been reported to occur in up to 30% of patients, with some variation according to ethnicity and geographical distribution.^5^^,^^6^ Previous studies and a recent meta-analysis suggest that individuals with seronegative disease typically present with predominantly renal-limited disease and experience poorer outcomes despite being generally younger with fewer comorbidites.^6^^,^^7^ The reasons for this remain unclear and may relate to delays in diagnosis and treatment in the context of negative serology, although it has also been proposed that ANCA-negative vasculitis represents a distinct spectrum of disease altogether.

As a relatively small subgroup of a rare disease, seronegative patients have been poorly represented in clinical trials; moreover, observational studies evaluating the clinical phenotype, treatment, and outcomes of this patient group have been limited, leaving numerous unanswered questions. This study aims to investigate the clinical presentation, histopathological findings, treatment practices, and outcomes of patients with ANCA-negative PIGN compared with ANCA-positive controls.

## Methods

### Patient Cohort

A retrospective cohort study was undertaken with data collected from 15 International centers and registries (Innsbruck, Austria; Zagreb, Croatia; Prague, Czech Republic; Munich, Germany; Chapel Hill, USA; Madrid, Barcelona, Spain; Brescia, Italy; London, Reading, Cambridge, Manchester, Preston, and Salford, UK; and the Irish registry). Patients with PIGN diagnosed from January 2002 to December 2022 were considered for inclusion.

The inclusion criteria comprised a diagnosis in those aged ≥18 years of small vessel systemic vasculitis according to the European Medicines Agency^8^ criteria with kidney involvement and biopsy-proven PIGN. Seronegative patients were required to be ANCA-negative on immunofluorescence and enzyme-linked immunosorbent assay at presentation and throughout the study period. A control cohort of ANCA-positive patients with either MPO-ANCA or PR3-ANCA positivity was recruited alongside with a target to achieve a 1:1 ratio. To ensure recruitment across the same timeframe, we aimed to enroll the next patients with MPO- and PR3-ANCA-positive PIGN diagnosed after an ANCA-negative PIGN case to serve as controls. Anti-MPO and anti-PR3 titers were defined as positive as per local standards. Patients without a kidney biopsy, nondiagnostic biopsy, or biopsy findings of significant dense deposits on electron microscopy were excluded. In addition, patients with antiglomerular basement membrane antibody positivity, evidence of other immune-mediated glomerular pathology, or patients with secondary vasculitis as a result of infection, drugs, or malignancies were excluded.

Data, including demographics, antibody status, clinical presentation, treatment and clinical outcomes were retrospectively collected from patient records. Histopathological findings were extracted from biopsy reports provided by the included centers. The outcomes of primary interest were all-cause mortality, ESKD, and relapse rates.

### Definitions

ESKD was defined using continued kidney replacement therapy (KRT) or transplantation at follow-up. Disease relapse was defined as a change in signs and symptoms that were attributed to vasculitis and required the reintroduction or increase in immunosuppressive treatment. Disease remission was defined as the absence of disease activity. Kidney function was estimated using the standardized Chronic Kidney Disease Epidemiology Collaboration formula (ml/min per 1.73 m^2^).^9^ PIGN was defined by the presence of crescents and necrotizing glomerulonephritis on light microscopy, with few or no immune deposits by immunofluorescence and few or no electron dense deposits on electron microscopy.^10^ Arteries and arterioles were also evaluated for the presence of vasculitis as well as the degree of interstitial fibrosis and tubular atrophy.

This study was approved and compliant with individual centers’ local ethic committees and Health Research and Innovation centers. Data were collected and analyzed using data sharing agreements and following the Caldicott principles. For those included from the RITA-Ireland registry, informed consent for their data to be used was obtained as part of participants’ inclusion within the registry.

### Statistical Analysis

Data analyses were conducted using R statistical software v4.1.2 (R Foundation, Vienna, Austria). We generated summary statistics across the above characteristics as follows: binomial and categorical characteristics were compared using proportions, continuous characteristics were compared using averages (means for symmetrically distributed data and medians for skewed data) and measures of dispersion (SDs and interquartile ranges [IQRs], respectively). Significance testing was carried out by using logistic regression for binomial traits; categorical traits were composed into binomial traits and then tested with logistic regression; continuous traits by linear regression. Missing data were excluded and the regressions were calculated with the complete case method. Kaplan-Meier plots were used in survival analyses of time to death and the difference between groups was compared with the log-rank test.

When we compared ANCA-positive with ANCA-negative patients, differences in characteristics between the groups, such as age, may have caused the differences in outcomes, rather than differences in the disease process. Alongside unadjusted analyses, we also used inverse probability weighting (IPW) to estimate the effect of the ANCA-negative PIGN process independent of these other characteristics. This technique has been used in causal inference with observational studies to compare interventions.^11^ We calculated the probability of each ANCA-negative patient having ANCA-negative disease and vice versa, based on the patient’s age, sex, ethnicity and recruiting center. This was done using a logistic regression model. We then performed a weighted regression of ANCA category on patient outcomes. The weight for each patient is the reciprocal of the previously calculated probability. This means that patients with a very high probability of either having ANCA-negative or ANCA-positive disease based on their previous characteristics have a reduced weight in the regression, and vice versa. This has the effect of balancing these confounders between the groups and allows a better estimate of the true causal effect of having ANCA-negative PIGN. We extended the IPW analysis to control for severity of disease—estimated with 2 variables, continuous serum creatinine at presentation and a binary variable of ESKD at diagnosis—and choice of induction treatment, estimated with 5 binary variables for steroid use generally, cyclophosphamide, rituximab, methylprednisolone specifically, and/or the use of plasma exchange.

## Results

### Baseline Characteristics and Clinical Presentation

Data for 132 ANCA-negative and 127 ANCA-positive patients (PR3-ANCA: *n* = 62; MPO-ANCA: *n*=65) with PIGN were included in the final analysis (Table 1). Patients were diagnosed across 2 decades, with similar diagnosis dates between the cohorts (Supplementary Figure S1). Owing to missing data, ANCA-positive patients were included from 5 international centers (Chapel Hill, USA; London, Cambridge, Preston, UK; and Munich, Germany). The mean age of ANCA-negative patients was 56.6 ± 17 years compared with ANCA-positive patients who had a higher mean age of 63.6 ± 14.5 years (*P* < 0.001). The ethnicity of patients included were predominantly White; 118 (89.4%) ANCA-negative versus 109 (85.8%) ANCA-positive. There were less Black and minority ethnic patients in the ANCA-negative cohort comparatively; 8 (6%) versus 16 (12.6%) but more Hispanic (*n* = 6 [4.6%] vs. *N* = 2 [1.6%], respectively).

**Table 1:** Baseline characteristics and outcomes of patients with ANCA-negative versus ANCA-positive pauci-immune glomerulonephritis

Baseline characteristics and outcomes	ANCA-negative (*n* = 132)	ANCA-positive (*n* = 127)	Unadjusted *P*-value
Age, yrs (mean ± SD)	56.6 ±17.0	63.6 ±14.5	<0.001
Sex, male: female (*n*)	68:64	72:55	0.44
ANCA type; MPO:PR3	-	65:62	-
Serum creatinine, μmol/l (median, IQR)	354.5 (198–639)	231.0 (141–354)	<0.001
eGFR, ml/min (median, IQR)	14 (7–30)	19 (12–40)	0.02
UPCR mg/mmol, (median, IQR)	347 (68–1067)	184 (93–391)	0.01
Organ system involvement, *n* (%)			
Constitutional Symptoms	65 (49.2)	67 (52.8)	0.57
Cutaneous	34 (25.8)	23 (18.1)	0.14
Mucocutaneous/Ophthalmic	6 (4.5)	9 (7.1)	0.39
ENT	12 (9.1)	45 (35.4)	< 0.001
Respiratory	22 (16.7)	46 (36.2)	< 0.001
Cardiovascular	3 (2.3)	5 (3.9)	0.44
Abdominal	3 (2.3)	6 (4.7)	0.29
CNS	6 (4.5)	14 (11.0)	0.06
Renal-limited	69 (52.3)	39 (30.7)	< 0.001
KRT at presentation, (%)	47 (35.6)	22 (17.3)	0.07
Relapse rates, (%)	12 (12.1%)	47 (37%)	< 0.001
ESKD at 1 yr, (%)	36 (27.7)	11 (8.7)	< 0.001
ESKD at 3 yrs, (%)	42 (33.3)	15 (13.0)	< 0.001
Mortality at 1 yr, (%)	12 (9.1)	5 (3.9)	0.09
Mortality at 3 yrs, (%)	15 (11.4)	14 (11.0)	0.96
Follow-up duration, mo (median, IQR)	80 (46–145)	98 (53–138)	0.63

ANCA, antineutrophil cytoplasmic autoantibodies; CNS, central nervous system; eGFR, estimated glomerular filtration rate (ml/min per 1.73 m^2^); ENT, ears, nose, and throat; ESKD, end-stage kidney disease; IQR, interquartile range; KRT, kidney replacement therapy; MPO, anti-myeloperoxidase; PR3, anti-proteinase 3, UPCR; urine protein creatinine ratio.

ANCA-negative patients had lower rates of ear, nose, and throat; and respiratory involvement compared with ANCA-positive controls; 9.1% versus 35.4%, and 16.7% versus 36.2%, (*P* < 0.001) respectively. Less than 5% of the ANCA-negative cohort had ophthalmic, cardiovascular, abdominal, or central nervous system involvement. Significantly more ANCA-negative patients presented with renal-limited disease (52.3% vs. 30.7%, *P* < 0.001) and they presented with a lower median estimated glomerular filtration rate (14 ml/min per 1.73 m^2^ [IQR: 7–30] vs. 19 ml/min per 1.73 m^2^ [IQR: 12–40], *P* = 0.02).Owing to limitations in adjustment methods, we used overlapping confidence intervals as the basis for comparing the phenotype of ANCA-negative, MPO-ANCA, and PR3-ANCA groups. The clinical characteristics of ANCA-negative and MPO-ANCA patients appeared more similar than when comparing ANCA-negative and PR3-ANCA positive patients (Supplementary Figure S2a).

One hundred eighteen ANCA-negative and 101 ANCA-positive patients had urine protein-to-creatinine ratio results available at presentation. ANCA-negative patients presented with higher levels of proteinuria (347 mg/mmol [IQR: 68–1067] vs. 184 mg/mmol [IQR: 93–391], *P* = 0.01).

### Histological Characteristics

The histological characteristics are presented in Table 2. The median percentage of cellular and fibrocellular crescents seen in ANCA-negative patients was similar to that in the ANCA-positive cohort (30% vs. 33.3%, *P* = 0.44). There was a higher median percentage of sclerosed glomeruli (11.1%) compared with ANCA-positive patients (8%), although this was not significant (*P* = 0.26).

**Table 2:** Histological characteristics of patients presenting with ANCA-negative versus ANCA-positive pauci-immune glomerulonephritis

Characteristics	ANCA-negative (*n* = 132)	ANCA-positive (*n* = 127)	*P*-value
Total number glomeruli (mean, SD)	18.7 ±9.4	20.2 ± 10.8	0.25
Normal glomeruli, (%)	34.6	35.9	0.78
Crescentic glomeruli, (%)	35.0	37.7	0.44
Sclerosed glomeruli, (%)	17.4	14.8	0.26
IFTA, *n* (%)			
None	21 (15.9)	28 (22.0)	0.21
Mild	40 (30.3)	33 (26.0)	0.44
Mild–moderate	32 (24.2)	42 (33.1)	0.11
Moderate	22 (16.7)	20 (15.7)	0.84
Moderate - severe	7 (5.3)	2 (1.6)	0.10
Severe	10 (7.6)	2 (1.6)	0.02
Extra glomerular arteritis, *n* (%)	10 (7.6)	12 (9.5)	0.59
Vessel wall necrosis, *n* (%)	24 (18.2)	12 (9.5)	0.04
Berden Classification,^12^*n* (%)			
Crescentic	36 (27.3)	42 (33.1)	0.92
Focal	38 (28.8)	37 (29.1)	0.43
Mixed	48 (36.4)	40 (31.5)	0.36
Sclerotic	10 (7.6)	8 (6.3)	0.70

ANCA, antineutrophil cytoplasmic autoantibodies; IFTA, interstitial fibrosis and tubular atrophy.

ANCA-negative patients presented with more interstitial fibrosis and tubular atrophy with a higher number of patients demonstrating moderate to severe (5.3% vs. 1.6%) and severe (7.6% vs. 1.6%) interstitial fibrosis and tubular atrophy. Features of extraglomerular arteritis was similar between the groups; however, there was a significantly higher percentage of vessel wall necrosis present in those with ANCA-negative PIGN (18.2% vs. 9.5%, *P* = 0.04). There was no significant difference in histological features according to the Berden classification.^12^

### Remission Induction Therapy

The remission induction treatments are outlined in Table 3. ANCA-negative patients were treated more often with cyclophosphamide induction (*n* = 82, 66.1% vs. *n* = 60, 47.6%; *P* = 0.02) and ANCA-positive patients received more combination treatment with rituximab and low dose cyclophosphamide together (*n* = 50, 39.7% vs. *n* = 22, 17.7%; *P* < 0.001). Induction with rituximab was used overall less frequently but with a similar percentage between the groups (12.1% vs. 11.1%). In those who were treated with cyclophosphamide, there was a higher cumulative cyclophosphamide dosage in the ANCA-positive cohort (5.64 ± 3.31 g vs. 4.15 ± 3.37 g; *P* = 0.11).

**Table 3:** Remission induction and maintenance treatments for patients with ANCA-negative versus ANCA-positive pauci-immune glomerulonephritis

	ANCA-negative (*N* = 132)	ANCA-positive (*N* = 127)	*P* value
Induction treatment, *n* (%)	124 (93.9)	126 (99.2)	0.23
i.v. methylprednisolone, *n* (%)	85 (68.5)	63 (50)	0.02
i.v. methylprednisolone, g (mean ± SD)	1.46 ± 0.87	1.41 ± 0.99	0.67
Cyclophosphamide, *n* (%)	82 (66.1)	60 (47.6)	0.02
Cyclophosphamide dosage, g (mean ± SD)	4.15 ± 3.37	5.64 ± 3.31	0.11
Rituximab, *n* (%)	15 (12.1)	14 (11.1)	0.91
Combination therapy with rituximab and Cyclophosphamide, *n* (%)	22 (17.7)	50 (39.7)	< 0.001
Alternative therapies,^a^*n* (%)	5 (4.0)	2 (1.6)	0.19
Plasma exchange, *n* (%)	39 (31.5)	32 (25.4)	0.37
Plasma exchange sessions, median (IQR)	6 (5–7)	7 (5–7)	-
Maintenance treatment, *n* (%)	107 (82.3)	119 (92.4)	0.02
Rituximab, *n* (%)	13 (12.1)	46 (38.7)	< 0.001
Azathioprine, *n* (%)	65 (60.7)	68 (57.1)	0.58
MMF, *n* (%)	18 (16.8)	23 (19.3)	0.62
Corticosteroid monotherapy, *n* (%)	17 (15.9)	13 (10.9)	0.29
Corticosteroid duration, months, median (IQR)	16 (3–41.3)	32 (11.5–78.5)	0.001

ANCA, antineutrophil cytoplasmic autoantibodies; IQR, interquartile range; IV, intravenous, MMF; mycophenolate mofetil.

a Alternative induction therapies included MMF, azathioprine, and corticosteroid monotherapy.

Four patients were treated with corticosteroid monotherapy; 3 ANCA-negative and 1 ANCA-positive. Pulsed i.v. methylprednisolone was used more frequently in ANCA-negative patients, but the mean dose was similar between the groups (*P* = 0.67). Other alternative induction therapies in the ANCA-negative group included mycophenolate mofetil (*n* = 1) and azathioprine (*n* = 1). Plasma exchange was performed more frequently in ANCA-negative patients (31.5% vs. 25.4%), although this was not statistically significant (*P* = 0.37).

### Maintenance Therapy

Fewer ANCA-negative patients received ongoing maintenance therapy than those with positive ANCA serology (*n* = 107, 82.3% vs. *n* = 119, 92.4%; *P* = 0.02), although when only looking at patients who have survived beyond 1 year, there was no significant difference (92.2% vs. 95.2%; *P* = 0.23).

Eleven ANCA-negative patients (10.3%) compared with 27 (22.7%) ANCA-positive patients received ≥ 2 maintenance immunosuppressive treatments during follow-up. Rituximab was used less frequently as a maintenance treatment in those with ANCA-negative PIGN (*n* = 13 vs. *n* = 46) (Table 3). Azathioprine was used similarly across both ANCA-negative and ANCA-positive cohorts (*n* = 65 vs. *N* = 68, respectively) as was mycophenolate mofetil (*n* = 18 vs. *n* = 23, respectively).

Corticosteroid as maintenance monotherapy therapy was used in 17 ANCA-negative patients and 13 ANCA-positive patients. The median duration of corticosteroid treatment was analyzed in 217 patients. ANCA-positive patients (*n* = 124) had a longer duration of corticosteroid treatment with a median of 32 (IQR: 11.5–78.5) months than the ANCA-negative cohorts (*n* = 93) who received a much shorter median duration of 16 (IQR: 3–41.3) months (*P* = 0.001).

### Kidney Outcomes

Forty-seven ANCA-negative patients (35.6%) required KRT at diagnosis compared with 22 ANCA-positive patients (17.3%) (*P* = 0.07). Of the 47 ANCA-negative patients requiring KRT, 15 (31.9%) recovered independent kidney function after treatment and 3 of these patients later went on to develop ESKD. In comparison, 11 of ANCA-positive patients (50%) requiring KRT at diagnosis recovered independent kidney function after treatment and only 1 patient subsequently developed ESKD.

From the total cohort of 132 ANCA-negative patients, 42 (31.8%) progressed to ESKD at 3 years, in comparison with 15 out of 127 patients (11.8%) with ANCA-positive PIGN. When comparing clinical outcomes, IPW to control for age, sex, ethnicity, and recruiting center was applied. ANCA-negative patients had significantly higher rates of ESKD at 1 year and 3 years (*P* < 0.001, respectively). Further analysis showed ESKD at 1 and 3 years remained significant when adjusted for the type of induction therapy (*P* < 0.001) but not when accounting for the severity of disease at presentation (*P* = 0.1) (Supplementary Table S1). Logistic regression of ANCA-negative patients was carried out to determine factors which could influence the outcome of ESKD. This included age, sex, ethnicity, treating center, induction therapy, dialysis dependence, and serum creatinine at presentation. We found that serum creatinine at presentation (odds ratio = 1.003/μmol/l, *P* < 0.001) correlated with increased risk of ESKD at 1 year. Treatment with plasma exchange also correlated with increased risk of ESKD at 1 year (odds ratio = 3, *P* = 0.01), although this is likely confounded by indication.

Sensitivity analysis was carried out in the 5 centers with ANCA-positive controls and there remained a significant difference in ESKD at 1 and 3 years (*P* ≤ 0.001) (Supplementary Table S2). Similar to affected organ systems, overlapping confidence intervals suggest ANCA-negative and MPO-ANCA positive patients have more comparable outcomes than ANCA-negative and PR3-ANCA positive patients (Supplementary Figure S2b).

### Mortality Outcomes

**Figure 1:**
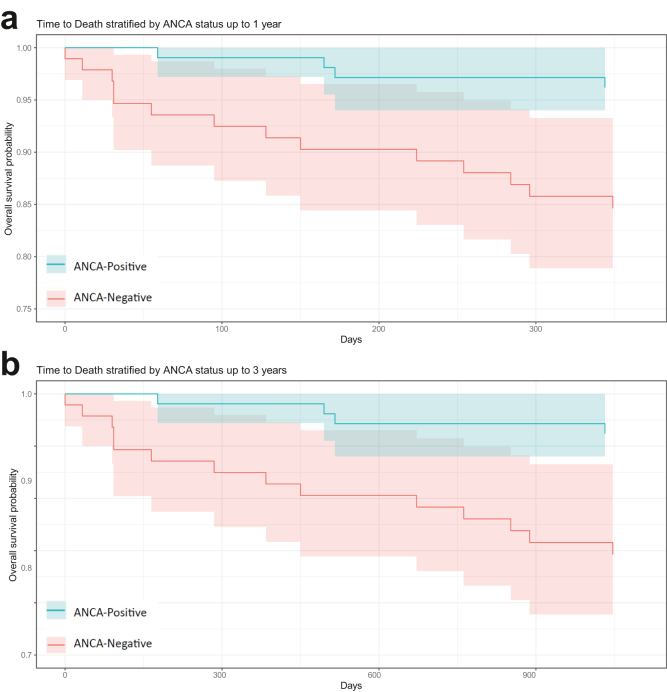
(a) Kaplan-Meier curve demonstrating the overall survival probability of patients with pauci-immune glomerulonephritis stratified by ANCA status up to 1 year from diagnosis. (b) Kaplan-Meier curve demonstrating the overall survival probability of patients with pauci-immune glomerulonephritis stratified by ANCA status up to 3 years from diagnosis.

Fifteen ANCA-negative (11.4%) and 14 ANCA-positive patients (11%) died within 3 years of diagnosis. When applying IPW for demographic variables there was an increased risk of death in the ANCA-negative cohort at 1 year (*P* = 0.01), shown in Figure 1a, but no statistical difference seen at 3 years (*P* = 0.21) (Figure 1b). The Kaplan Meier curve visually diverged in the first 10 years of follow-up because of a higher percentage of censored patients in the ANCA-negative cohort (Supplementary Figure S3). Death at 1 year reverted to a nonstatistically significant increase in the ANCA-negative group when severity of disease (*P* = 0.21) or induction treatment choice (*P* = 0.57) was controlled for (Supplementary Table S1). The overall survival analysis of time to death showed no significant difference between cohorts by log-rank test (*P* = 0.70). Most patients with a documented cause of death died from infections (*n* = 10), other causes included congestive cardiac failure (*n* = 1), interstitial lung disease (*n* = 1) and liver failure (*n* = 1). In 16 patients, the cause of death was unknown. The mean age at the time of death was lower in the ANCA-negative group (65 ± 12.8 years) compared with the ANCA-positive cohort (72 ± 11.4 years) (*P* < 0.001).

### Relapse

Relapse rates were lower in ANCA-negative patients (*n* = 12 [12.1%] vs. *n* = 47 [37%], *P* < 0.001), and time to relapse was shorter in ANCA-positive controls; 23 ± 21.4 months compared with 50.6 ± 40 months, respectively. When considering patients who were not in kidney failure at diagnosis, the risk of relapse was still lower in the ANCA-negative cohort (*P* < 0.001). Further sensitivity analysis was carried out to account for those treated with corticosteroid maintenance monotherapy, and relapserates remained lower in the ANCA-negative group (12% vs. 22%, *P* ≤ 0.001). We also adjusted for the severity of disease and induction treatment choice. In all circumstances, relapse rates remained significantly lower in ANCA-negative patients than in ANCA-positive patients (*P* < 0.001).

## Discussion

This multicenter retrospective study found that patients with ANCA-negative PIGN are characterized by lower estimated glomerular filtration rate’s, more frequently require KRT at presentation, and have worse overall outcomes than patients with ANCA-positive PIGN. This is despite being younger and aged below the typical peak incidence age, expected in the sixth and seventh decade of life. Our findings highlight distinct differences in clinical features, outcomes, and treatment approaches of ANCA-negative PIGN when compared with those with seropositive disease. This study corroborates the findings of a recent meta-analysis involving 301 patients with ANCA-negative, confirming similar clinical presentations, demographics, and outcomes.^7^ However, the inclusion of ANCA-positive comparators and treatment data offers new insights into the disease process.

Within our cohort, ANCA-negative patients predominately exhibited renal-limited disease with fewer extrarenal symptoms despite its classification as a systemic disease. Similar findings have been reported in MPO-ANCA positive patients who more often present with kidney impairment and lower rates of relapse.^1^ When comparing the phenotype and outcomes of our ANCA-negative cohort, we observed greater similarities between those with MPO-ANCA positivity, than those with PR3-ANCA positivity. Previous research by Eisenberger *et al.* demonstrated histological parallels between MPO-ANCA and ANCA-negative cases, revealing greater levels of fibrosis and chronic kidney disease than in PR3-ANCA-positive patients.^13^ In addition, Roth *et al.* identified restricted autoantibody responses in ANCA-negative disease and demonstrated that specific MPO-ANCA epitopes are associated with pathogenic properties and disease activity, suggesting shared mechanisms between ANCA-negative disease and MPO-autoantibodies.^14^ This raises the question of whether ANCA-negative patients are truly ANCA-negative or if other circulating autoantibodies have the potential for neutrophil activation which are not detectable via conventional assays.^7^ Furthermore, Eisenberger *et al.* also demonstrated that neutrophil infiltration and glomerular lesions can occur irrespective of circulating ANCA,^13^ suggesting alternative immune mechanisms, autoantibodies, and epitope diversity may contribute.^7^^,^^15^^,^^16^

Delays in diagnosis may contribute to more severe disease at presentation resulting in histopathological findings indicative of chronic damage, as well as higher levels of proteinuria. Although we showed numerically more severe interstitial fibrosis and tubular atrophy and glomerulosclerosis at presentation in patients with ANCA-negative PIGN, the number of glomerular lesions did not differ and there was no statistical difference overall. Previous studies report varied histological findings in ANCA-negative PIGN with some, similar to ours, finding no significant difference in chronicity,^7^^,^^17^ whereas others observed more extensive fibrosis.^13^^,^^18^ Some published cases report heavy proteinuria and nephrotic syndrome associated with ANCA-negative PIGN,^6^^,^^17^^,^^19^ which may stem from mesangial and endocapillary proliferation or the possibility of a podocyte defect that is unique to seronegative PIGN.^17^ These factors likely explain the poorer kidney outcomes seen in ANCA-negative PIGN especially because proteinuria is a known risk factor for chronic kidney disease.

We demonstrated a significantly higher rate of ESKD at 1 and 3 years in those with ANCA-negative PIGN, similar to findings reported in other studies.^6^^,^^7^^,^^20^ This was supported by the IPW analysis, which showed that this effect was removed by controlling for severity of disease at presentation, implying that long-term ESKD outcomes can be explained by the severity of kidney disease at presentation. We also observed an increased mortality risk for ANCA-negative patients within the first year although this was not appreciated at 3-year follow-up. Infection remained the leading cause of death despite patients receiving less intense immunosuppressive treatment.

When considering remission induction and maintenance therapy, 8 ANCA-negative patients did not receive induction treatment. Four of these patients were dialysis-dependent at presentation and the increased risk of serious infection associated with dialysis may explain the decision to withhold immunosuppressive therapy in these cases.^21^^,^^22^ Induction with cyclophosphamide was more common in ANCA-negative PIGN, with fewer patients receiving combination (cyclophosphamide and rituximab) therapy. ANCA-positive cohorts received higher cumulative doses of cyclophosphamide and longer duration of corticosteroids. Despite the absence of circulating autoantibodies, more ANCA-negative patients received plasma exchange. This was associated with an increased risk of ESKD at 1 year and likely confounded by indication, because ANCA-negative patients presented with significantly higher serum creatinine and lower estimated glomerular filtration rate, reflecting more severe kidney disease at presentation. These treatment differences suggest that ANCA-negative PIGN is managed differently by clinicians, with immunosuppression often initiated earlier in ANCA-positive cases. This disparity may stem from uncertainty around initiating treatment in the absence of ANCA or extrarenal manifestations.

Notably, there was a significantly lower rate of relapse in patients with ANCA-negative PIGN even when accounting for those receiving KRT. This has been shown in a previous study^23^ and may suggest the potential for shorter duration of maintenance treatment in seronegative patients, especially in view of their largely renal-limited disease. This is of importance because ANCA-negative patients have been poorly represented in clinical trials and newer therapeutic approaches have been mostly assigned to ANCA-positive patients. In the absence of a prospective clinical trial, treatment practices, protocols, and guidelines have been inferred from the data available for seropositive PIGN and may not reflect the optimal treatment for those with ANCA-negative disease.

The current diagnostic classification systems for vasculitis are not without limitations especially when considering ANCA-negative disease. The Chapel Hill Consensus Conference criteria,^24^ the European Medicines Agency criteria,^8^ and the more recent 2022 ACR/EULAR classification criteria^25^, ^26^, ^27^ have all been developed over the years for the purpose of aiding diagnosis and facilitating clinical research. The current Diagnostic and Classification Criteria in Vasculitis group has developed new classification criteria with ANCA being accorded substantial significance as a diagnostic marker. In a patient presenting with renal-limited PIGN in the absence of detectable ANCA, they do not meet the required points for a classification of microscopic polyangiitis or granulomatosis with polyangiitis.^26^^,^^27^ In contrast, the European Medicines Agency criteria does not require ANCA-positivity to fulfil the entry criteria and definitions.^28^ It is therefore likely that ANCA-negative disease is underrepresented because of the limitations of these diagnostic algorithms.^29^

Limitations of this study include the retrospective design. Only patients with kidney involvement were included and the clinical characteristics of ANCA-negative vasculitis in patients without PIGN were not considered. Missing data resulted in ANCA-positive patients being included from only 5 centers, which has the potential to confound results; however, we performed sensitivity analysis to account for this. Plasma exchange was identified as a risk factor for ESKD; however, this is likely to be confounded by indication and more accurately reflects the more severe kidney disease of ANCA-negative patients at diagnosis. The lack of standardized treatment recommendations for ANCA-negative patients complicates comparisons, and not all patients had complete data on cumulative treatment doses. Assessing the time-to-diagnosis would have been important for determining if there were delays in diagnosis and what effect this had on outcomes; however, this data was not available, and analysis not performed. Although the cohort size may be underpowered to detect significant differences between the cohorts, it is the largest study of its kind. Furthermore, though improved international standards and newer generations of enzyme-linked immunosorbent assay tests have reduced the potential for false negatives,^30^ current assays may still miss some epitopes and differences across the centers have the potential to confound results. Nevertheless, ensuring patients were ANCA-negative throughout the duration of their disease should overcome any center differences or changes of the assays overtime.

## Conclusion

Patients with ANCA-negative PIGN have worse outcomes than patients with ANCA-positive PIGN. Although the exact pathophysiology underlying seronegative PIGN and factors contributing to its clinical phenotype remain unclear, the adverse outcomes may be attributed to diagnostic uncertainties, leading to treatment hesitancy in the absence of positive serology. A lack of robust biomarkers and limitations in current diagnostic and classification criteria inadequately support patients with ANCA-negative disease. Current treatment recommendations are based largely on studies involving ANCA-positive patients and there is a pressing need for collaborative research as well as the inclusion of ANCA-negative patients in clinical trials.

## Disclosure

LF has received speaking fees from CSL Vifor. MAL received an unrestricted research grant from CSL Vifor. US has received study fees and consultancy fees from Alexion/AstraZeneca, Ablynx/Sanofi, and Chemocentryx/Vifor; and lecture fees from Janssen-Cilag, Alexion/AstraZeneca, Sanofi, and Vifor. ZH and VT have received consulting and speaking fees from CSL Vifor and AstraZeneca. VKD has received consultancy fees from Novartis, Forma Therapeutics, Travere Therapeutics, and Amgen; and has received royalties from UpToDate. NB received speaking and consultancy fees from CSL Vifor and Boehringer Ingelheim plus support for meeting registration and travel from CSL Vifor. LFQ received consultancy and speaking fees from CSL Vifor, Novartis, Otsuka, and GSK. SRB has received consultancy and speaker fees from CSL Vifor. DG received consulting fees from Amgen, Calliditas, Vera Therapeutics, Sana Biotech, GSK, Outsuka, and Aurinia Inc. SPM has received research grants from AstraZeneca &TheriniBio; and consultancy or speaker fees from CSL Vifor, GlaxoSmithKline, and Celltrion. AK received support for meeting registration and travel from AstraZeneca and Otsuka, an unrestricted research grant from CSL Vifor and consultancy fees or speaking fees from Amgen, AstraZeneca Boehringer Ingelheim, CSL Vifor, Delta4, GlaxoSmithKline, Miltenyi Biotech, Novartis, NovoNordisk, Roche, Sobi, and Walden Biosciences. AK is an editorial board member of NDT and Glomerular Diseases. All the other authors declared no competing interests.

## Supplementary Material

Supplementary File (PDF) **Figure S1.** Year of diagnosis of included patients with ANCA-negative and ANCA-positive pauci-immune glomerulonephritis. **Figure S2.** (a) Proportion of ANCA-negative, MPO-ANCA, and PR3-ANCA patients affected by different organ systems involvement with associated confidence intervals. (b) Outcomes associated with ANCA-negative, MPO-ANCA, and PR3-ANCA patients with associated confidence intervals. **Figure 3.** Kaplan-Meier curve demonstrating the overall survival probability of patients with pauci-immune glomerulonephritis stratified by ANCA status at the end of study. **Table S1.** Inverse probability weighting (IPW) table for 7 outcome variables by ANCA vasculitis status. **Table S2.** Sensitivity analysis conducted using data from the 5 recruiting centers that provided ANCA-negative patients and all the ANCA-positive patients as controls. STROBE Statement—Checklist of items that should be included in reports of cohort studies.

## Footnotes

Supplementary File (PDF)
**Figure S1.** Year of diagnosis of included patients with ANCA-negative and ANCA-positive pauci-immune glomerulonephritis. **Figure S2.** (a) Proportion of ANCA-negative, MPO-ANCA, and PR3-ANCA patients affected by different organ systems involvement with associated confidence intervals. (b) Outcomes associated with ANCA-negative, MPO-ANCA, and PR3-ANCA patients with associated confidence intervals. **Figure 3.** Kaplan-Meier curve demonstrating the overall survival probability of patients with pauci-immune glomerulonephritis stratified by ANCA status at the end of study. **Table S1.** Inverse probability weighting (IPW) table for 7 outcome variables by ANCA vasculitis status. **Table S2.** Sensitivity analysis conducted using data from the 5 recruiting centers that provided ANCA-negative patients and all the ANCA-positive patients as controls. STROBE Statement—Checklist of items that should be included in reports of cohort studies.
